# A pilot time-in-bed restriction intervention behaviorally enhances slow-wave activity in older adults

**DOI:** 10.3389/frsle.2023.1265006

**Published:** 2024-01-03

**Authors:** Kristine A. Wilckens, Rima F. Habte, Yue Dong, Michelle E. Stepan, Kibra M. Dessa, Alexis B. Whitehead, Christine W. Peng, Mary E. Fletcher, Daniel J. Buysse

**Affiliations:** ^1^School of Medicine, University of Pittsburgh, Pittsburgh, PA, United States; ^2^Department of Psychiatry, University of Pittsburgh Medical Center, Pittsburgh, PA, United States; ^3^School of Nursing, University of Pittsburgh, Pittsburgh, PA, United States

**Keywords:** behavioral slow-wave sleep enhancement, slow-oscillation, sleep restriction, memory retention, cognitive aging

## Abstract

**Introduction:**

Identifying intervention methods that target sleep characteristics involved in memory processing is a priority for the field of cognitive aging. Older adults with greater sleep efficiency and non-rapid eye movement slow-wave activity (SWA) (0.5–4 Hz electroencephalographic activity) tend to exhibit better memory and cognitive abilities. Paradoxically, long total sleep times are consistently associated with poorer cognition in older adults. Thus, maximizing sleep efficiency and SWA may be a priority relative to increasing mere total sleep time. As clinical behavioral sleep treatments do not consistently enhance SWA, and propensity for SWA increases with time spent awake, we examined with a proof-of concept pilot intervention whether a greater dose of time-in-bed (TiB) restriction (75% of habitual TiB) would increase both sleep efficiency and SWA in older adults with difficulties staying asleep without impairing memory performance.

**Methods:**

Participants were adults ages 55–80 with diary-reported sleep efficiency < 90% and wake after sleep onset (WASO) >20 min. Sleep diary, actigraphy, polysomnography (PSG), and paired associate memory acquisition and retention were assessed before and after a week-long TiB restriction intervention (*n* = 30). TiB was restricted to 75% of diary-reported habitual TiB. A comparison group of *n* = 5 participants repeated assessments while following their usual sleep schedule to obtain preliminary estimates of effect sizes associated with repeated testing.

**Results:**

Subjective and objective sleep measures robustly improved in the TiB restriction group for sleep quality, sleep depth, sleep efficiency and WASO, at the expense of TiB and time spent in N1 and N2 sleep. As hypothesized, SWA increased robustly with TiB restriction across the 0.5–4 Hz range, as well as subjective sleep depth, subjective and objective WASO. Despite increases in sleepiness ratings, no impairments were found in memory acquisition or retention.

**Conclusion:**

A TiB restriction dose equivalent to 75% of habitual TiB robustly increased sleep continuity and SWA in older adults with sleep maintenance difficulties, without impairing memory performance. These findings may inform long-term behavioral SWA enhancement interventions aimed at improving memory performance and risk for cognitive impairments.

## Introduction

Deeper, more consolidated sleep is consistently associated with better memory and cognitive outcomes in older adults (Wilckens et al., 2018). For instance, older adults with higher sleep efficiency and slow-wave activity (SWA) (0.5–4 Hz electroencephalographic (EEG) activity during non-rapid eye movement sleep) exhibit better cognitive function, memory recall (Wilckens et al., 2014, 2016, 2017), and overnight memory retention (Hokett et al., 2021). Moreover, SWA within the slow-oscillation range (0.5–1 Hz) is particularly implicated in consolidation of memories, cognitive health, and Alzheimer's disease pathology (Staresina et al., 2015; Mander et al., 2017; Mikutta et al., 2019). Thus, identifying sleep intervention methods that robustly enhance SWA, particularly the slow-oscillation, and are feasible to implement in older populations, is a top priority for the field of non-pharmacological interventions for cognitive aging and memory disorders.

Various SWA enhancement techniques, such as transcranial or acoustic stimulation have proven fruitful in young adults (Zhang and Gruber, 2019) and some studies in older adults with a single night or nap design have shown promise for enhancing SWA and associative memory (Westerberg et al., 2015; Papalambros et al., 2017). However, multiple meta-analyses and failed replication studies suggest these approaches have weaker and non-significant effects in older adults (Paßmann et al., 2016; Schneider et al., 2020; Wunderlin et al., 2021; Stanyer et al., 2022; Harlow et al., 2023). Stimulation approaches are further limited in their ecological validity, cost, and accessibility, due to their dependence on specialized equipment and potential to wake participants who have a low arousal threshold, such as older adults (Wilckens et al., 2018).

Behavioral interventions are promising because of their feasibility and ecological validity for long-term use. While a common misconception is that improved sleep involves increased sleep duration, long total sleep time (TST) is commonly associated with *poorer* cognition in older adults (Stone et al., 2009; Loerbroks et al., 2010; Devore et al., 2014; Wilckens et al., 2014). Whereas, more efficient sleep, high in slow-wave activity, tends to be associated with better cognition function in older adults (Anderson and Horne, 2003; Mander et al., 2013; Blackwell et al., 2014; Wilckens et al., 2014, 2016, 2017). Further, it is common for older adults, particularly in retirement, to sacrifice sleep depth for greater sleep time through excessive time in bed (TiB) and daytime naps (Frisoni et al., 1996; Ito et al., 2000). These sleep habits may increase sleep duration (Webb and Aber, 1984), but severely fragment sleep and minimize SWA (Frisoni et al., 1996; Ito et al., 2000).

Time-in-bed (TiB) restriction is one approach commonly used in behavioral interventions for insomnia, such as Cognitive Behavioral Treatment for Insomnia (CBTI) to enhance homeostatic sleep drive and enhance sleep efficiency (Borbély et al., 2016; Hogan et al., 2020). Paradoxically, TiB restriction may be more beneficial in older groups because it enhances sleep efficiency. Moreover, slight sleep restriction does not impair cognitive function to the same extent in older as young adults (Duffy et al., 2009; Landolt et al., 2012). Thus, a behavioral intervention that adjusts TST to a moderate duration while optimizing sleep efficiency and time spent in deeper stages of sleep may be beneficial to cognition in older adults.

Accordingly, we have found that among older adults undergoing Brief Behavioral Treatment for Insomnia (BBTI), those who have larger increases in SWA tend to have greater improvements in cognition (Wilckens et al., 2016). However, SWA changes are not consistent in behavioral treatments for insomnia. This may be due to differences in the extent to TiB restriction between participants and interventions. Various studies of sleep restriction therapy limit TiB to the duration of habitual TST, often including additional TiB, i.e., TiB = TST + 30 min (Kyle et al., 2015). Thus, in older adults with excessive wake time in bed, a greater dose of TiB restriction may reduce TiB spent awake and in lighter sleep stages. This may in turn increase not only sleep efficiency as found with conventional sleep treatments, but SWA as well.

There may be a “sweet spot” for TiB to maximize sleep drive to enhance SWA in older adults without decreasing time spent in deeper, more restorative stages of sleep, to prevent sleep-restriction-related impairments. Accordingly, one study in older adults with TiB > 8 h, 90 min of TiB restriction for 8 weeks revealed that more TiB restriction was associated with improved cognitive function (Youngstedt et al., 2009).

The current proof-of-concept experimental intervention study was conducted in older adults with difficulties staying asleep to examine whether SWA could be robustly enhanced in older adults with a feasible behavioral intervention. Through TiB restriction to 75% of each participant's habitual TiB, the current intervention was intended to maximize homeostatic sleep drive, at the expense of wake TiB and lighter stages of sleep, i.e., N1 sleep, while otherwise maintaining adequate sleep opportunity. We hypothesized that 1 week of TiB restriction to 75% of habitual TiB would lead to increases not only in sleep efficiency, but also in SWA, assessing both the slow oscillation (0.5–1 Hz) and delta activity (1–4 Hz). This level of restriction was chosen based on average TiB in our prior behavioral treatment studies using CBTI and BBTI, which did not robustly enhance either measure of SWA with ~85% TiB on average (Wilckens et al., 2016; Hogan et al., 2020). We examined primary measures of changes in TiB, TST, sleep efficiency, and WASO using diary, actigraphy, and PSG. We used quantitative NREM sleep EEG to assess changes in SWA within the slow-oscillation (0.5–1 Hz) and delta activity (1–4 Hz) ranges. As a preliminary proof-of-concept, we tested whether cued recall memory performance was impaired, unchanged, or improved with this level of TiB restriction. We secondarily examined participant-reporting of side effects, including sleepiness and sleepiness-related safety issues over the course of the week-long intervention.

## Materials and methods

### Participants

Participant characteristics are displayed in Table 1. Participants were older adults who reported difficulties staying asleep, ages 55–80 years. Participants were recruited from the community through a variety of methods including the University of Pittsburgh research participant registry, Craigslist, and advertisements. All participants provided informed consent in line with the University of Pittsburgh Institutional Review Board. We aimed to identify older adults with low sleep efficiency, such that their sleep would be improved with a TiB restriction intervention. Participants were included if they had < 90% sleep efficiency and >20 min of wake after sleep onset (WASO) based on a 1-week assessment of the Pittsburgh Sleep Diary (Monk et al., 1994), confirmed with actigraphy. Sleep efficiency for determining eligibility was defined based on the time of “lights out” to “final awakening” (Levenson et al., 2013). This stringent definition of sleep efficiency with a 90% cutoff has been shown to have the highest sensitivity and specificity for distinguishing good sleeping older adults from those with insomnia (Levenson et al., 2013). While we did not aim to specifically recruit participants with insomnia, twenty-two participants (62.8%) reported Insomnia Severity Index (ISI) scores ≥ 11 at baseline, suggesting over half the sample had clinical insomnia (Seow et al., 2018).

**Table 1:** Participant characteristics in the TiB restriction and comparison groups.

	**TiB restriction**	**Comparison**
*N*	30	5
Age	66.4 (7.4)	66.7 (4.8)
N Female	20 (66.7%)	4 (80%)
Race (*n* white)	24 (80%)	4 (80%)
*N* years of education	16.1 (2.3)	18.0 (5.5)
PSQI (month) total	9.5 (3.5)	8.4 (1.7)
AHI	5.5 (4.2)	6.5 (1.3)
*N* medications	3.4 (2.6)	3.2 (1.8)
*N* medical conditions	1.6 (1.0)	1.2 (1.8)

AHI, apnea hypopnea index. Parentheses indicate standard deviations, except for *N* female and race variables, which indicate % of participants in the sample.

**Figure 1:**
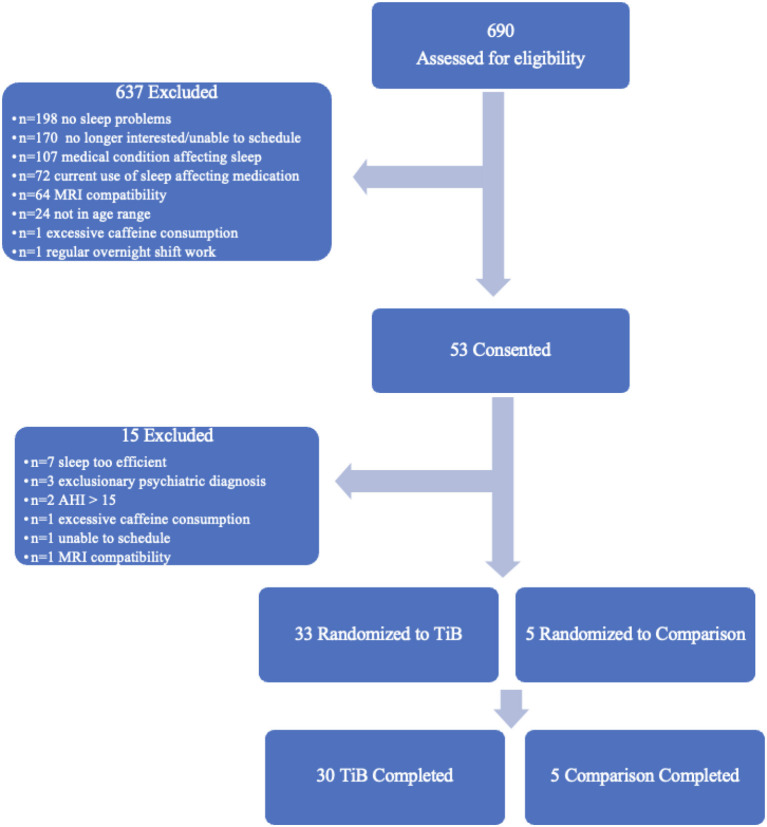
Participant flow chart. Six-hundred and ninety participants were assessed for eligibility. Fifty-three of these were consented and completed in-person eligibility assessments. Thirty-three participants were randomized to the TiB restriction intervention and five participants were randomized to the comparison group. Thirty TiB participants and five comparison participants completed study procedures.

Additional exclusion criteria were: a current sleep disorder other than insomnia, including restless legs syndrome, circadian rhythm disorder, and REM sleep behavior disorder, an apnea hypopnea index > 15 based on a single-night in-home Apnealink monitor, current or recent (within the past 6 months) shift work involving regular work after 9 p.m., medications that affect sleep such as antidepressants, anticonvulsants, steroids, or antipsychotic medications, current or recent severe psychiatric conditions or neurological disorders, consumption of >14 alcoholic beverages per week or >6 within one sitting or >3 caffeine drinks per day based on a 1-week diary. As this study included MRI, participants were additionally excluded if they were not eligible to complete an MRI scan (data not reported here). Medical and psychiatric exclusion was based on a brief neuropsychiatric interview administered by a trained clinician and a medical history and physical administered by a resident physician. The participant flow chart is displayed in Figure 1. Thirty-five participants completed all study procedures and were included in the present analyses. Thirty of these were randomized to an active TiB restriction group and 5 were randomized to a comparison group. Participants were randomized to the TIB restriction group at a rate of 4 out of 5 and were randomized to the comparison group at a rate of 1 out of 5. Analyses were focused on the TiB restriction group, and analyses in the comparison group were performed to preliminarily estimate repeated testing effects.

### Procedure

After telephone screening, participants came into the laboratory to complete informed consent, questionnaires, and were given an Apnealink device, a sleep diary, a daily Karolinska Sleepiness Scale, and an Actiwatch 2 device (Phillips Respironics) to take home for 1 week. Participants were given instructions to use the Apnealink for one night and to send it back in the mail. Participants learned to use the Apnealink device by watching an instructional video in the lab and through a demonstration from research staff. Participants were given instructions by staff for completing the sleep diary, sleepiness scale, and actigraphy assessments for seven consecutive days, and were asked to mail these supplies back. Eligibility was based on the in-home diary, confirmed with actigraphy, and the Apnealink. For enrolled participants, data from the diary, Actiwatch, and questionnaires were used as baseline (T1) data.

Participants eligible based on diary and apnea assessments were invited back for two in-laboratory polysomnography (PSG) assessments spaced 1 week apart (T1 and T2) including a paired associate cued recall overnight memory retention task and questionnaires of the Epworth Sleepiness Scale and the ISI. For the two overnight visits, participants arrived at the Sleep and Behavioral Neuroscience Center at University of Pittsburgh ~4 h before their designated in-bed time. Participants began the cued recall task within ~1 h of arrival. The interval between the end of the week-long baseline assessment and the first PSG night and the start of the intervention ranged between 1 and 4 weeks (mean = 1.8 weeks, sd = 0.8), depending on the availability of the participant for overnight PSG. During the week intervening the two PSG assessments, participants completed another sleep diary, daily Karolinska Sleepiness Scale ratings, and wore an Actiwatch as they followed instructions associated with the intervention. Participants who completed the study were asked to complete a post-study questionnaire to gain qualitative data on their subjective experience with the intervention.

### Intervention

TiB based on the baseline sleep diary was used to determine the sleep schedule for each participant undergoing the TiB restriction intervention. After the first PSG visit, participants began the 1-week intervention phase. All participants completed a sleep diary and wore the Actiwatch during the intervention phase.

Participants undergoing the TiB restriction intervention were asked to follow a sleep schedule that limited their TiB to 75% of their habitual TiB (average sleep opportunity including naps), truncated equally at the beginning and end of the night. For instance, if an individual was in bed for an average of 8 h from 11 p.m. to 7 a.m., they were assigned a TiB of 6 h with in- and out-of-bed times from 12 a.m. to 6 a.m. Sleep schedules had a minimum TiB of 5 h. One participant who would have had a prescribed TiB < 5 h was given a TiB equivalent to 79% of their habitual TiB. All participants were cautioned that sleepiness is a common side effect of TiB restriction and that if they felt sleepy, they should not drive or engage in any activities for which safety could be compromised due to sleepiness.

To preliminarily estimate effects of repeated testing in the absence of TiB restriction, a small set of participants (*n* = 5) was randomly assigned to a comparison intervention at a rate of one every five participants. The purpose of the comparison group was to obtain preliminary estimates of effect sizes of repeated testing, with the caveat that the small comparison sample precludes drawing definitive conclusions about repeated testing effects. Participants in the comparison group were provided a summary of their average sleep times, similar to the TiB restriction group and were asked to follow their typical/average in- and out-of-bed times as closely as possible. Comparison group participants were also asked to avoid taking naps during the 1-week intervention to account for potential circadian effects with eliminating daytime sleeping in the TiB restriction intervention.

Bachelor's level staff conducted daily check-in phone calls during the week of the intervention with both groups of participants to address questions, remind participants of the sleep schedule, assess sleepiness, and remind participants about sleepiness-related safety precautions. Participants reported daily sleepiness and in- and out-of-bed times from their diary at each check-in. If sleepiness was ≥8 on the Karolinska sleepiness scale for >2 days, participants were told not to engage in any activities where safety could be compromised due to sleepiness and TiB schedules would be adjusted. No adjustments needed to be made to the sleep schedule based on Karolinska sleepiness ratings for any participants. Participants were asked to report any adverse events including excessive sleepiness at each phone contact and were to call study staff to spontaneously report adverse events throughout the intervention.

### Diary data collection and processing

Participants were asked to complete their sleep diary twice a day: in the morning they reported on the previous nights' sleep and in the evening about their daytime activities. Diary variables examined were TiB, TST, sleep efficiency, WASO, sleep quality, subjective sleep depth, sleepiness at bedtime, and daytime alertness.

### Actigraphy data collection and processing

Actigraphy sleep data were collected on the Actiwatch 2 (Phillips Respironics) in 30 s epochs. Participants wore the Actiwatch on their non-dominant wrist. They were instructed to indicate with an event marker when they tried to go to sleep and when they got out of bed. Processing took place in Actiware version 6 set to medium sensitivity (defined as a Wake Threshold Value of 40.00). Scoring of actigraphy data was guided by the diary in- and out-of-bed times. Automatic detection of the sleep period was based on inactivity for at least ten consecutive minutes following the in-bed time, with less than one epoch of movement within those minutes. Sleep period end time was determined by the last epoch of a period of continuous activity. Sleep onset was identified as the time between the start of the rest period (in-bed to out-of-bed time) and start of the sleep period. TiB was defined as the total length of the rest period. TST was defined as the length of the sleep period. Sleep efficiency was defined as TST/TiB. WASO was define as the total number of minutes where activity was above the wake threshold during the sleep period.

### PSG data collection, processing, and analysis

In- and out-of-bed times for the PSG nights were based on the sleep diary averages at the first time point and based on the prescribed sleep schedule at the second time point. The PSG recording montage included F3, F4, C3, C4, O2, O1. F and C channels were processed and analyzed here. The sleep record was visually scored in 30-s epochs. Automated spectral analysis was performed on the EEG data using a 512-point fast-Fourier transform with epochs scored as NREM sleep (Vasko et al., 1997). Absolute and relative power were calculated in 0.5 Hz bins for each channel, F3, F4, C3, C4, across all of NREM sleep. Absolute power reflected the area under the curve of total power within each 0.5 Hz bin. Relative power was calculated by dividing the total power within 0.5 Hz bin by the total power in the 0.5–32 Hz range. Relative power was tested to account for potential individual differences in total EEG power. Epochs identified as artifact by automated algorithm (Brunner et al., 1996) and visual inspection were rejected. Data from the 0.5–1 Hz and 1–4 Hz bands were examined in the present analysis. We analyzed only NREM sleep SWA because SWA is highest during NREM sleep.

PSG sleep variables were TiB, defined as the amount of time spent between going into bed and getting out of bed; TST, defined as the amount of time spent sleeping combining REM sleep and NREM sleep together; sleep efficiency, defined as TST/TiB; and WASO, defined as the amount of time spent awake after sleep onset.

### Paired associate cued recall task

The paired associate recall task consisted of face-profession pairs. Two versions of the task were designed for each of the two time points, counterbalanced across participants. An encoding and immediate recall test took place in the evening before the overnight sleep study. A delayed recall test took place the next morning after the overnight sleep study. For the encoding task, twenty face-profession pairs were presented one at a time on a computer screen and participants were asked to envision the person pictured carrying out the duties of the profession appearing in text below the face image. The encoding task involved participants rating on a scale of 1–3 how difficult it was to form the mental image. Participants were then given an immediate cued recall test whereby they were shown each face and asked to recall out loud the profession paired with the face displayed. The experimenter typed in the profession to ensure accurate spelling. Participants were required to correctly recall 70% of the pairs at test to ensure adequate encoding. On incorrect trials, feedback was immediately given showing the correct profession below the face. If participants did not reach 70% criterion, the set of 20 recall trials was repeated until they reached 70% criterion (≥14 professions correctly recalled) or until the 10th recall cycle. The delayed recall test administered the next morning after the overnight sleep study was identical to the immediate test. Two measures: accuracy and number of cycles were assessed for immediate and delayed recall tests. To assess paired associate acquisition, number of cycles was used as the dependent variable. To assess overnight memory retention, accuracy at the final immediate cycle and the first delayed cycle were compared.

### Statistical analyses

In line with the proof-of-concept nature of this study, primary analyses focused on effects of time point for each sleep measure in the TiB restriction group. Effects of time point were also tested in the comparison group to estimate of effect sizes of repeated testing in the absence of TiB restriction. We examined the significance of time-point effects in the TiB restriction group and compared effect sizes (mixed model parameter estimates in the units of the individual variables) to those in the comparison group. In line with an intent-to-treat approach, all participants were included regardless of adherence to the intervention. To ensure all data were valid, actigraphy data were excluded for time points with < 4 days of actigraphy data (*n* = 2 at baseline, and *n* = 1 at intervention) and PSG nights with unusable data due to excessive artifact were excluded (*n* = 2 at T1 and *n* = 2 at T2). Follow-up procedures were not completed for actigraphy in *n* = 1 and PSG in *n* = 1.

Linear mixed models using a compound symmetry covariance matrix were run to test for effects of time point as a repeated measure for each sleep measure. Mixed model parameter estimates and standard errors were included for all effects of time point on sleep. Parameter estimates were used as the estimate of effect size in the TiB restriction group relative to the comparison group. Participant ID was included in analyses as a random effect and timepoint was a fixed effect repeated measure. For spectral measures, channel (F3, F4, C3, C4) was also included as a fixed effect repeated measure. No time point × channel interactions were significant. Therefore, all analyses below included all channels (F3, F4, C3, C4), controlling for channel as a within subject fixed effect repeated measure.

Analysis structure for the paired associate cued recall memory task was similar to those conducted for the sleep measures. Linear mixed models tested for effects of time point and test type (immediate or delayed recall) for the two recall measures (number of cycles to reach criterion and retention). Analyses controlled for task version. Participant ID was included as a random effect and time point, test type, and version were included as fixed effect repeated measures.

Secondary measures included sleepiness over the course of the intervention assessed daily with the Karolinska sleepiness scale. We examined sleepiness ratings each day of the TIB restriction intervention relative to sleepiness ratings each day at baseline (Supplementary Figure 1). Linear mixed model analyses were run separately in the TiB restriction and comparison groups. Time point (baseline, intervention) and Day (1–7) were repeated measures. Karolinska sleepiness rating (scale of 1–10) was the dependent variable. Additional main effects of time point were assessed in both groups for the Epworth Sleepiness Scale, ISI, PSG-assessed sleep stages, and diary measures of sleep quality, sleep depth, sleepiness at bedtime, daytime alertness, daytime mood, and daytime naps. Finally, a post-study questionnaire was administered in interview format to obtain feedback from participants on their subjective experience with the sleep schedule they were prescribed and how they thought their sleep and cognitive abilities changed with the intervention. These results are described below in terms of the proportion of participants reporting a given type of feedback in each group.

## Results

**Figure 2:**
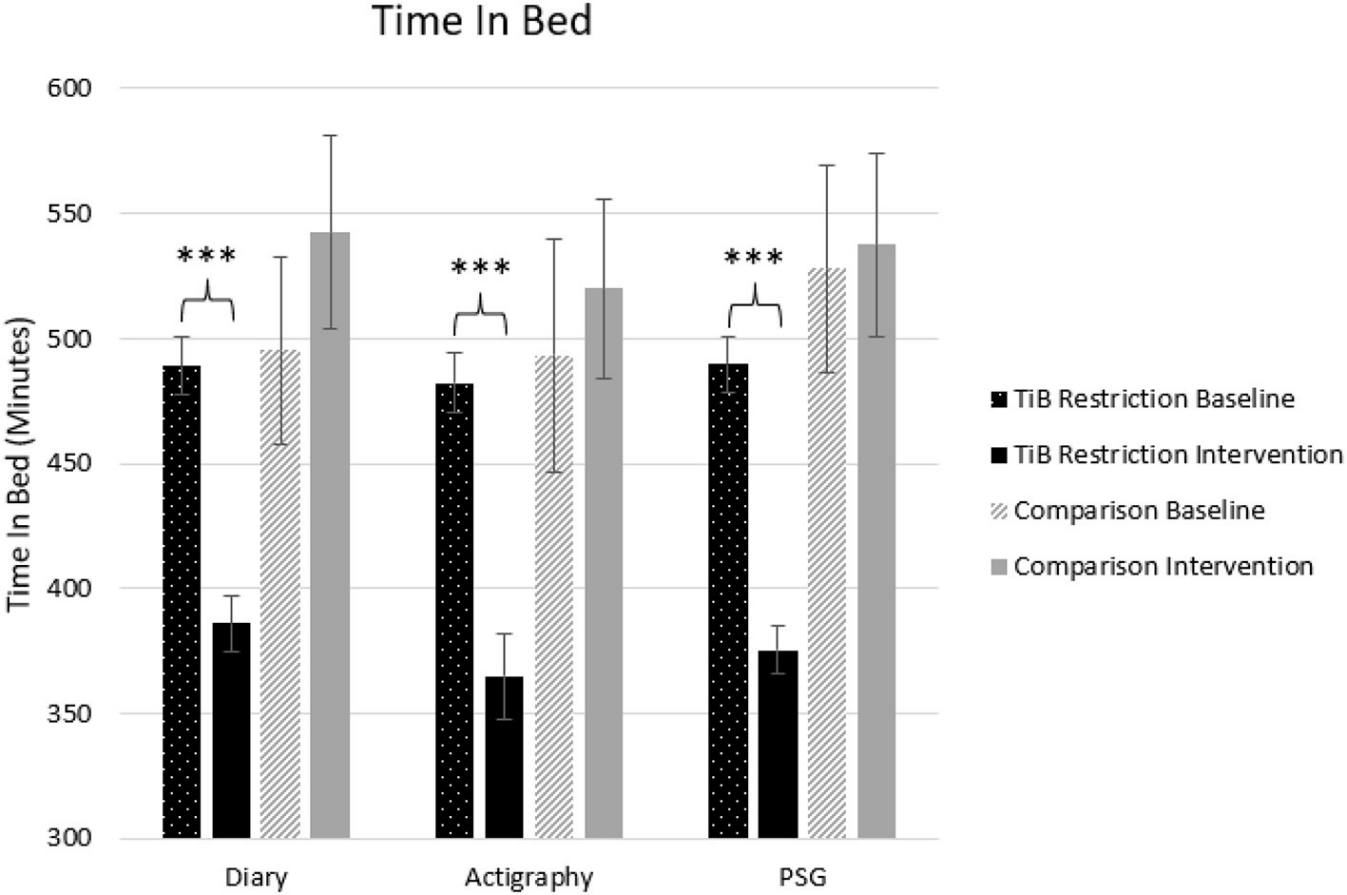
Mean TiB for diary, actigraphy, and PSG at baseline and during the intervention for the TiB restriction and comparison groups. TiB restriction showed significant decreases in TiB, with non-significant effects in the opposite direction for the comparison group. Asterisks denote significant effects of time point within group (***p < 0.001). Error bars indicate standard errors.

**Figure 3:**
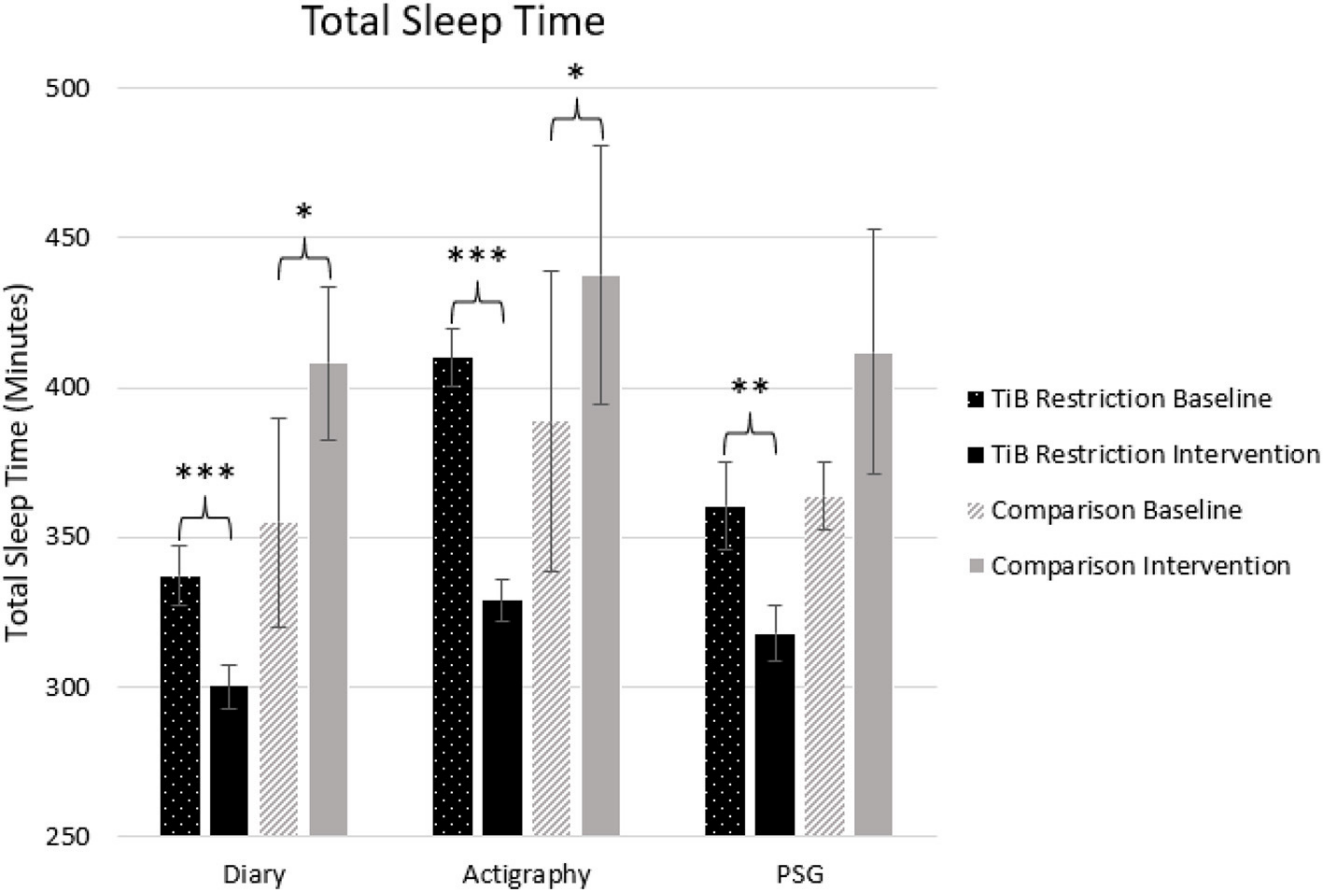
Mean TST for diary, actigraphy, and PSG at baseline and during the intervention for the TiB restriction and comparison groups. TiB restriction showed significant decreases in TST across measures. The comparison group showed a significant increase in TST for diary and actigraphy. Asterisks denote significant effects of time point within group (*p < 0.05, **p < 0.01, ***p < 0.001). Error bars indicate standard errors.

**Figure 4:**
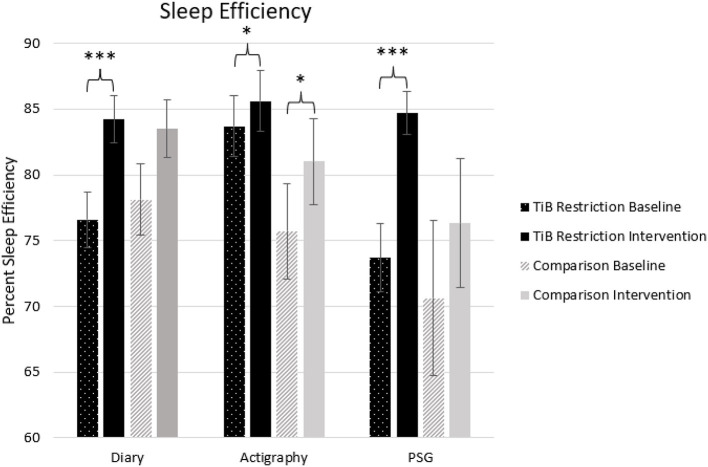
Mean sleep efficiency for diary, actigraphy, and PSG at baseline and during the intervention for the TiB restriction and comparison groups. TiB restriction exhibited improvements in diary and PSG sleep efficiency. Both groups exhibited significant increases in actigraphy sleep efficiency. Asterisks denote significant effects of time point within group (*p < 0.05, ***p < 0.001). Error bars indicate standard errors.

**Figure 5:**
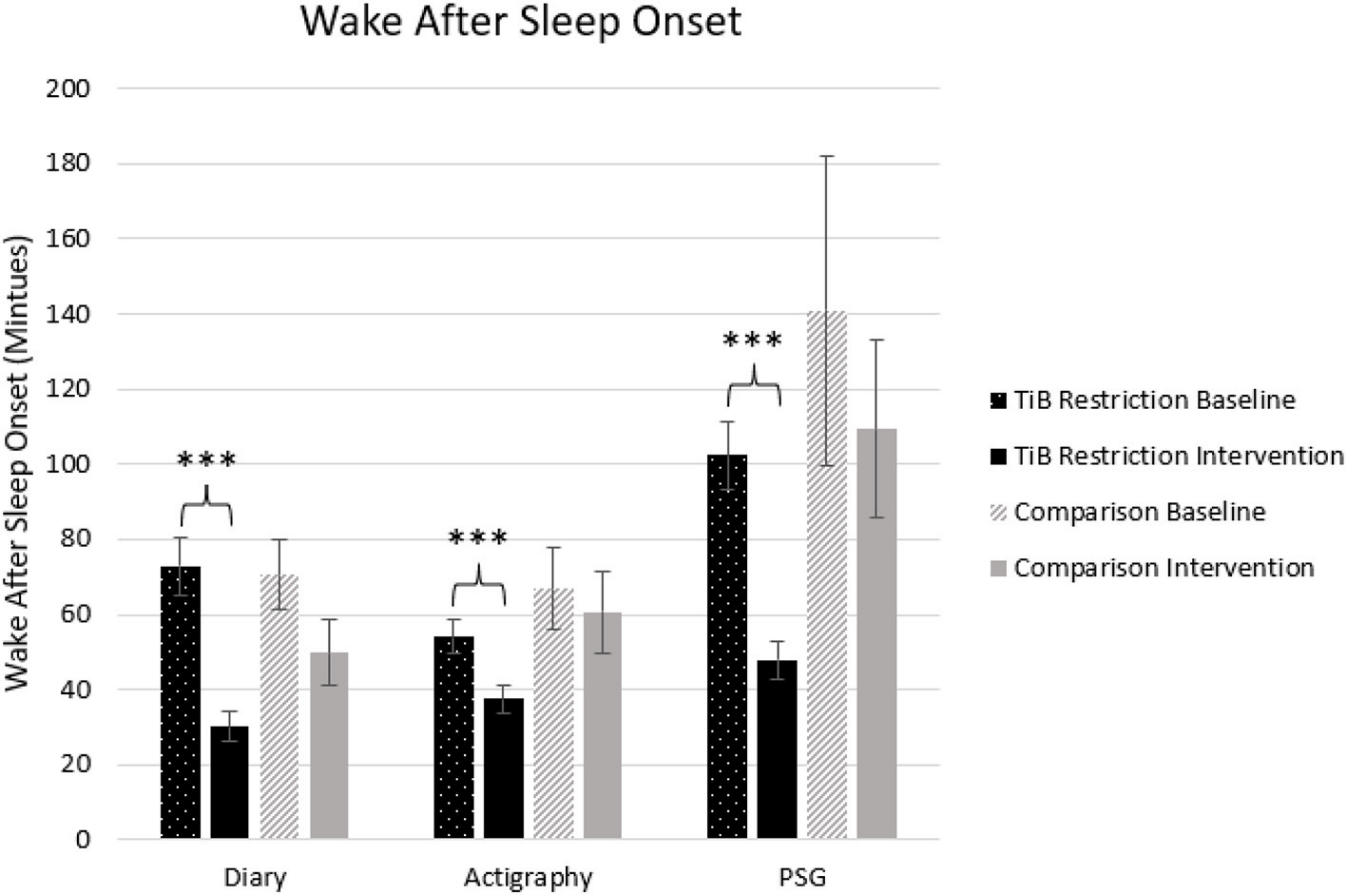
Mean WASO in minutes for diary, actigraphy, and PSG at baseline and during the intervention for the TiB restriction and comparison groups. TiB restriction uniquely showed significant decreases in WASO across diary, actigraphy and PSG. Non-significant and consistently smaller effects in the same direction were found the comparison group. Asterisks denote significant effects of time point within group (***p < 0.001). Error bars indicate standard errors.

**Figure 6:**
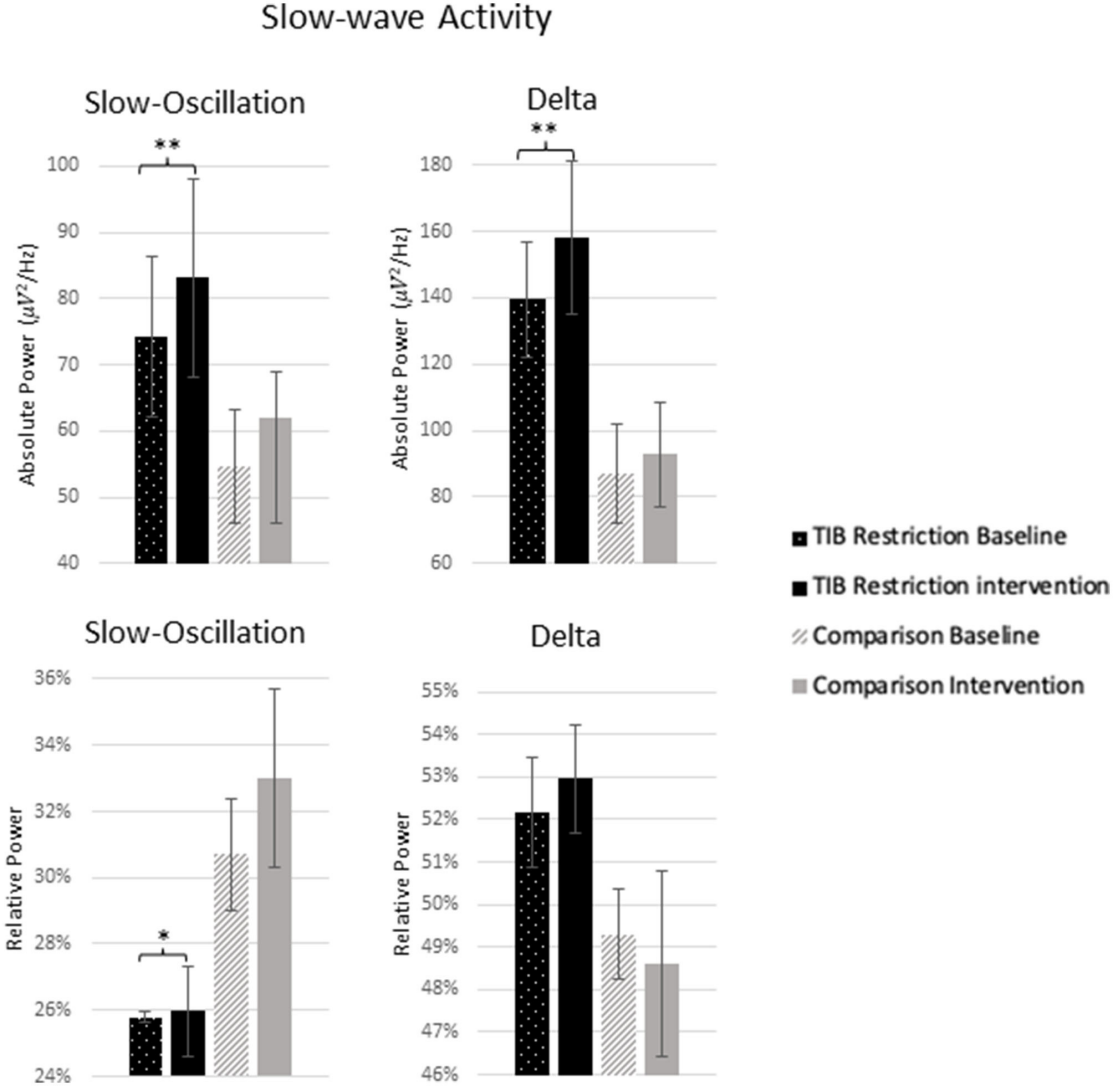
Mean absolute and relative power for 0.5–1 Hz (slow-oscillation) range and 1–4 Hz (delta) range at baseline and following the intervention for the TiB restriction and comparison groups. TiB restriction group showed significant increase in absolute spectral power for 0.5–1 and 1–4 Hz, and relative spectral power for 0.5–1 Hz. Asterisks denote significant effects of time point within group (*p < 0.05, **p < 0.01). Error bars indicate standard errors.

Within-group change statistics are presented in Table 2 for primary (TiB, TST, sleep efficiency, and WASO, and SWA spectral power) and secondary sleep measures (questionnaires, reported naps, and PSG-assessed sleep stages). Karolinska sleepiness ratings are presented in the Supplementary material. Means and standard deviations for primary and secondary sleep measures are presented in Supplementary Table 1. Figures 2–6 display the results for primary sleep measures.

**Table 2:** Statistics for change in primary and secondary sleep measures for TiB and comparison groups.

	**TiB restriction group**					**Comparison group**				
	***F***	**df**	***p***	**Estimate**	**se**	***F***	**df**	***p***	**Estimate**	**se**
Epworth	0.5	1,28.2	0.468	0.4	0.5	0.01	1,4	0.947	0.2	2.9
**ISI**	**7.6**	**1,28.4**	**0.010**	**−1.7**	**0.6**	1.0	1,4	0.382	−1.6	1.6
**TiB Diary**	**126.8**	**1,29.0**	**< 0.001**	**−102.9**	**9.1**	5.3	1,4	0.083	47.2	20.5
**TiB Act**	**54.1**	**1,27.3**	**< 0.001**	**−115.8**	**15.8**	3.3	1,4	0.142	26.9	14.8
**TiB PSG**	**462.5**	**1,28.2**	**< 0.001**	**−115.2**	**5.4**	0.9	1,4	0.407	9.4	10.2
**TST Diary**	**25.4**	**1,29.0**	**< 0.001**	**−36.9**	**7.3**	**15.5**	**1,4**	**0.017**	**53.1**	**13.5**
**TST Act**	**95.8**	**1,26.0**	**< 0.001**	**−79.1**	**8.1**	**9.2**	**1,4**	**0.039**	**49.2**	**16.2**
**TST PSG**	**10.7**	**1,28.9**	**0.003**	**−42.4**	**13.0**	1.1	1,4	0.350	48.1	45.4
**SE Diary**	**23.8**	**1,29.0**	**< 0.001**	**7.7**	**1.6**	2.0	1,4	0.229	5.3	3.8
**SE Act**	**4.5**	**1,25.9**	**0.044**,	**2.1**	**0.97**	**8.1**	**1,4**	**0.046**	**5.3**	**1.9**
**SE PSG**	**17.7**	**1,28.9**	**< 0.001**	**11.1**	**2.6**	0.4	1,4	0.550	5.8	8.8
**WASO Diary**	**39.6**	**1,29.0**	**< 0.001**	**−42.6**	**6.8**	1.8	1,4	0.249	−20.6	15.3
**WASO Act**	**20.6**	**1,25.9**	**< 0.001**	**−16.4**	**3.6**	3.9	1,4	0.120	−6.3	3.2
**WASO PSG**	**44.7**	**1,28.8**	**< 0.001**	**−54.8**	**8.2**	0.4	1,4	0.573	−31.5	51.4
**Nap Mins**	**5.0**	**1,29.0**	**0.033**	**−32.2**	**14.4**	4.9	1,4	0.092	−328.4	148.5
**N1 Min**	**13.3**	**1,28.2**	**0.001**	**−9.8**	**2.7**	0.3	1,4	0.589	5.2	8.9
**N2 Min**	**7.7**	**1,29.0**	**0.009**	**−26.7**	**9.6**	0.7	1,4	0.451	27.5	32.9
N3 Min	0.1	1,28.3	0.753	−2.0	6.1	0.3	1,4	0.634	1.7	3.3
REM Min	0.6	1,28.5	0.459	−3.8	5.0	0.3	1,4	0.589	13.7	23.4
% N1	3.7	1,28.7	0.064	−2.4	1.3	0.03	1,4	0.875	0.5	2.7
% N2	0.01	1,28.8	0.947	−0.1	1.6	0.02	1,4	0.887	−0.5	3.0
% N3	0.8	1,28.3	0.380	1.3	1.5	0.0	1,4	0.989	−0.02	1.1
% REM	1.2	1,28.3	0.286	1.2	1.1	0.0	1,4	0.997	0.01	4.1
**Abs .5–1 Hz**	**7.6**	**1,201.3**	**0.007**	**10.5**	**3.8**	2.5	1,31	0.121	7.4	4.6
**Abs 1–4 Hz**	**9.2**	**1,201.4**	**0.003**	**17.0**	**5.6**	2.1	1,31	0.158	5.7	3.9
**Rel .5–1 Hz**	**5.1**	**1,201.0**	**0.025**	**0.010**	**0.004**	2.6	1,31	0.116	0.02	0.02
Rel 1–4 Hz	1.4	1,201.1	0.232	0.003	0.002	0.5	1,31	0.507	−0.007	0.01

Bold font denotes significant changes pre to post intervention. Mixed model estimate refers to the effect size for the main effect of time point in the units of the individual measures. Negative estimate values suggest a decrease. Act, Actigraphy; Min, Minutes; Abs, Absolute; Rel, Relative.

### Primary measures

#### Time in bed

Consistent with the intervention instructions, TiB decreased for the restriction group but not the comparison group. TiB significantly decreased for diary, actigraphy, and PSG measures. Time point effects sizes were smaller and not significant in the comparison group for diary, actigraphy, or PSG (Table 2; Figure 2).

#### Total sleep time

As hypothesized, the TiB restriction group showed significant decreases in TST for each measure, diary, actigraphy, and PSG. There was a significant increase in TST in both the diary and actigraphy for the comparison group. A numerical increase in PSG TST in the comparison group was not significant (Table 2; Figure 3).

#### Sleep efficiency

The TiB restriction group showed larger effect sizes and significant increases in sleep efficiency for diary, actigraphy, and PSG. Additionally, the comparison group showed an unexpected increase in sleep efficiency for actigraphy (Table 2; Figure 4).

#### Wake after sleep onset

The TiB restriction group uniquely showed significant decreases in WASO for diary, actigraphy, and PSG. Time point effect sizes were smaller and not significant for diary, actigraphy, and PSG in the comparison group (Table 2; Figure 5).

#### Slow-wave activity

The TiB restriction group exhibited increases in SWA for absolute slow-oscillation and delta activity. Changes in absolute SWA were not significant in the comparison group and effect sizes were smaller (Table 2; Figure 6).

The TiB restriction group additionally exhibited a significant increase in relative slow-oscillation power. However, a non-significant increase of similar effect size was also found in the comparison group. Relative delta power change was not significant in either group.

### Secondary sleep measures

#### Questionnaires

Karolinska sleepiness findings are presented in Supplementary Figure 1. Karolinska sleepiness scale ratings in the TiB restriction group significantly increased during the intervention week relative to the baseline week, *F*_(1, 355.5)_ = 8.9, *p* = 0.003. Follow-up paired samples *t*-tests demonstrated that increased sleepiness during the intervention relative to baseline was significant on Day 2, *t*_(27)_ = 2.1, *p* = 0.046 and Day 3, *t*_(25)_ = 2.4, *p* = 0.027, was marginally significant on Day 4, *t*_(25)_ = 2.02, *p* = 0.054, but was not significant on Day 1, *t*_(23)_ = 1.4, *p* = 0.182 or on the Days 5–7, (*p*-values > 0.28). Note that there was no significant main effect of Day, *F*_(7, 353.7)_ = 1.2, *p* = 0.283 or a time × day interaction, *F*_(7, 353.4)_ = 0.3, *p* = 0.956. For the Epworth sleepiness scale, there was no significant change for either group (Table 2). For the ISI, the TiB restriction group showed a significant decrease in insomnia severity (Table 2).

#### PSG-assessed sleep stages

Changes in minutes and percentage of each sleep stage are presented in Table 2. The TiB restriction group showed significant decreases in minutes of *N*1 and *N*2, while minutes of *N*3 were unchanged. The comparison group showed no significant changes by sleep stage.

#### Additional sleep diary measures

The TiB restriction group exhibited significant increases in subjective sleep quality, *F*_(1, 29)_ = 28.1, *p* < 0.001, estimate = 12.3, se = 2.3, sleep depth, *F*_(1, 29)_ = 18.6, *p* < 0.001, estimate = 13.3, se = 3.1, and sleepiness at bedtime, *F*_(1, 29)_ = 14.2, *p* < 0.001, estimate = 9.0, se = 2.4. The TiB restriction group further exhibited a marginal decrease in daytime alertness, *F*_(1, 29)_ = 2.9, *p* = 0.100, estimate = 5.7, se = 3.4. Subjective mood after final awakening as measured by the sleep diary did not show any significant changes in the TIB restriction group, *F*_(1, 29)_ = 2.4, *p* = 0.134, estimate = 2.7, se = 1.8. As instructed, the TIB group significantly reduced their total minutes spent napping (Table 2).

In contrast, the comparison group showed no significant changes in these additional sleep diary measures. Subjective sleep quality, *F*_(1, 4)_ = 2.2, *p* = 0.209, estimate = 10.3, se = 6.9, and sleep depth, *F*_(1, 4)_ = 1.1, *p* = 0.356, estimate = 9.7, se = 9.3, showed a similar pattern of numerical increases with smaller effect sizes relative to the TiB restriction group. Sleepiness at bedtime showed no change, *F*_(1, 4)_ = 0.0, *p* = 0.976, estimate = 0.2, se = 6.3, and daytime alertness non-significantly increased, *F*_(1, 4)_ = 1.5, *p* = 0.292, estimate = 8.3, se = 6.8. Subjective mood after final awakening showed no change in the comparison group, *F*_(1, 4)_ = 1.5, *p* = 0.292, estimate = 8.7, se = 7.2. As instructed, the comparison group also reduced their time spent napping, with a marginally significant reduction in total nap minutes (Table 2).

### Paired associate memory recall

For associative memory acquisition (number of cycles to reach criterion of 70% correct recall), the TiB group showed a main effect of test type such that they took fewer cycles to reach criterion at delayed test compared to immediate test, *F*_(1, 84.97)_ = 238.76, *b* = −4.24, se = 0.27, *p* < 0.001. The TiB group also showed a main effect of time point, such that the TiB group took, on average, fewer cycles to reach criterion at both immediate and delayed test at T2 compared to T1. *F*_(1, 85.59)_ = 5.60, (*b* = −0.66, se = 0.28, *p* = 0.020). The comparison group also showed the same main effect of test type, *F*_(1, 12)_ = 22.36, *b* = −3.30, SE = 0.70, *p* < 0.001, with fewer cycles at delayed compared to immediate, but no effects of time point *F*_(1, 12)_ = 1.3, *b* = 0.83, se = 0.71, *p* = 0.265.

To assess overnight memory retention, we compared accuracy at the final cycle of immediate test in the evening to accuracy at the first cycle of the delayed test the next morning. Both the TiB restriction and comparison groups showed a main effect of test type, TiB: *F*_(1, 84.95)_ = 44.79, (*b* = −0.12, SE = 0.02, *p* < 0.001), comparison: *F*_(1, 12)_ = 5.14, (*b* = −0.07, SE = 0.03, *p* = 0.043), suggesting significant forgetting from immediate in the evening to delay the next morning. There were no main effects or interactions with time point for overnight memory retention.

### Post-study questionnaire qualitative findings

94.3% (*n* = 33) participants completed the post-study questionnaire. Of those participants, twenty-nine were in the TiB restriction group and four were in the comparison group. Participants in the TiB restriction group endorsed having a positive experience with the intervention and eleven participants (38%) in the TiB restriction group stated that they would follow a similar schedule after the study was over. Twelve additional TiB participants (41%) stated that they would follow a similar schedule but with slightly less TiB restriction. A greater proportion of the TiB restriction group endorsed that their quality of sleep improved (89.7%) relative to the comparison group (50%). Participants (41%) in the TiB restriction group additionally reported being more sleepy than usual. Four participants in the TiB restriction group (14%) endorsed feeling that their sleepiness was potentially dangerous, and these four participants avoided driving during the TiB intervention in line with intervention safety instructions. Two additional TiB participants reported avoiding driving during the intervention due to sleepiness. Only one participant in the TiB restriction group felt that their cognitive function improved. Nine participants in the TiB restriction group felt that their cognitive function worsened (31%).

## Discussion

Clinical behavioral sleep treatments such as CBTI do not consistently or robustly modify aspects of sleep architecture such as SWA, which may contribute to their limited utility for improving cognition, memory, and risk for neurodegenerative disease (Wilckens et al., 2016, 2017; Perrault et al., 2022). Here we tested an experimental TiB restriction intervention focused on a higher dose of TiB restriction to enhance SWA at the expense of TST and lighter stages of sleep. We found that TiB restriction to 75% of the habitual time-in-bed led to robust improvements in sleep efficiency and WASO at the expense of time spent in stages *N*1 and *N*2. As hypothesized, TiB restriction was associated with significant increases in SWA across the entire SWA range (0.5–4 Hz), particularly for absolute power, suggesting a robust increase in homeostatic sleep drive.

In contrast to our prior work which demonstrated weak and inconsistent increases in the 0.5–1 Hz (slow-oscillation) range following CBTI in a larger sample of patients with chronic insomnia disorder (Hogan et al., 2020), the current intervention demonstrated consistent increases in absolute power within both the 0.5–1 Hz and 1–4 Hz range. This is noteworthy, as the slow-oscillation (0.5–1 Hz) and delta activity (1–4 Hz) are thought to be functionally distinct in promoting memory consolidation and their risk for memory disorders (Staresina et al., 2015; Winer et al., 2019). The slow oscillation in particular, is shown to be involved in memory consolidation and inversely associated with Alzheimer's disease pathology (Mander et al., 2017). Here we show that a greater dose of TiB restriction that impinges on lighter stages of NREM sleep to increase homeostatic sleep drive more robustly modulates the full range of SWA, including the slow-oscillation and delta activity.

It is further noteworthy that for relative power, only slow-oscillation activity increased in the intervention group, but delta power did not. While there has been little research in humans testing the dissociable role of the slow-oscillation and delta activity in tracking homeostatic sleep drive, our prior work in older adults with insomnia also showed that delta power defined as 1–4 Hz (absolute or relative) was sensitive to neither insomnia status nor CBTI. Relative slow-oscillation power however, was sensitive to insomnia and marginally increased after CBTI (Hogan et al., 2020). In contrast, a study of topographic changes in SWA with sleep deprivation in young adults (Bersagliere et al., 2018) showed that delta activity (1.25–2 Hz), but not slow oscillation activity (0.5–1 Hz) was sensitive to sleep deprivation. Age and insomnia status may be an important factor in these dissociable effects.

The lack of change in relative delta activity may further speak to the memory functions that may be sensitive to a TiB restriction intervention. Rodent and human studies suggest that slow-oscillations and their coordination with spindles are thought to be involved in strengthening of memories, whereas, delta activity is hypothesized to promote forgetting (Kim et al., 2019). While both strengthening and weakening of memories are essential for optimal memory function (Stickgold and Walker, 2013), greater delta activity, in contrast to slow-oscillation activity, has also been associated with poorer memory and greater memory-related pathology (Mander et al., 2015). Nonetheless, few studies in humans have examined these dissociable oscillations of SWA. Comprehensive examinations of slow-oscillation, delta activity, as well as their coordination with spindles is needed to understand how slow wave and delta oscillations interact to influence memory in the context of a behavioral intervention that manipulates homeostatic sleep drive.

Improvements were found across measures of sleep continuity (diary, actigraphy, and PSG) as well as subjective sleep depth. However, there was a reduction in TST, suggesting TiB restriction did not only reduce time spent in bed awake while trying to sleep. Nonetheless, the only significant reductions in time spent in each sleep stage were found in lighter stages of NREM sleep, *N*1 and *N*2—not *N3*, slow-wave sleep or REM sleep, which also plays a critical role in memory processing (Girardeau and Lopes-Dos-Santos, 2021). Thus, it is arguable that the intervention preserved components of sleep considered most restorative and critical for daytime function. A reduction in *N*2 could conceivably interfere with the benefits of spindle activity to memory consolidation, as this stage exhibits the greatest amount of spindle activity measured with EEG. However, spindle activity is present during *N*3 sleep as well, and the coupling of spindles with slow-waves, which are predominant during *N*3 sleep, is considered critical for consolidation processes (Staresina et al., 2015).

The cued recall task, which was intended to preliminarily test the acute effects of this level of TiB restriction on memory performance, showed similar levels of paired associate memory acquisition and overnight retention for the TiB restriction group at T1 and T2, with a small effect size improvement in acquisition. We interpret this as support for the proof-of-concept that TiB restriction to 75% does not appear to impair memory performance in older adults with low sleep efficiency. However, this study was not designed to be a comprehensive test of the hypothesis that a behavioral TiB restriction intervention leads to improved memory. These findings are intended to inform the optimization of longer-term and larger follow-up clinical trials to determine whether there are long-term memory improvements with such a behavioral intervention once participants adapt to the sleep schedule and sleepiness dissipates. It remains to be determined whether a similar intervention over several weeks would lead to memory improvements through enhanced slow oscillation and sleep efficiency at the expense of sleep time in lighter stages of NREM sleep.

## Limitations and future directions

While the current proof-of-concept study has implications for enhancing aspects of sleep that are most beneficial to memory, sleepiness was a major side effect of the intervention, and has substantial negative effects on various processes involved in memory including encoding and controlled retrieval (Wilckens et al., 2012). Nonetheless, prior reports demonstrate that the effects of TiB restriction on sleepiness and daytime function tend to be greatest within the first week of sleep restriction therapy treatments as participants adapt to the prescribed sleep schedule (Kyle et al., 2011; Maurer et al., 2018). In line with this, only one participant in the TiB restriction group endorsed feeling that their cognitive function improved but one third of participants in the TiB restriction group endorsed feeling that their cognitive function worsened. Sleepiness and daytime function, tend to improve at follow-up after 4 weeks of sleep restriction therapy (Kyle et al., 2011). In the current study, significantly elevated sleepiness was no longer significant in later days of the intervention relative to baseline. Thus, it is plausible that participants would further adapt to the sleep schedule with a longer intervention and the effects of improved sleep efficiency and SWA could lead to improvements in cognition and memory processing in the long-term. Conventional sleep restriction therapy and CBTI approaches typically extend TiB over the course of the intervention. Therefore, it is necessary to understand how the current dose of TiB restriction influences the trajectory of daytime function and long-term memory impairments as well as overall brain health.

As a proof-of-concept pilot study, this current sample size was very small. In particular, the comparison group was too small to draw conclusions beyond preliminary estimates of effect sizes for repeated testing. Thus, caution is highly warranted in interpreting any effects in this group. There were several measures in which the comparison group exhibited numerical improvements, which may have been due to the instructions to follow a consistent sleep schedule, elimination of daytime naps, or a first night effect. However, the high level of heterogeneity in older adults' sleep (Wallace et al., 2022) is unlikely to be captured in this sample of *n* = 5, and therefore these effects would need to be replicated in a larger sample to draw such conclusions. Moreover, interpretation of the unique intervention effects in the PSG data are further limited by the lack of an adaptation night in both groups. Future studies will benefit from more rigorous experimental control to determine the unique effects of the TiB restriction intervention independent of repeated assessments. Another limitation of the small sample size is that it precluded exploratory analyses of moderating factors such as age. The current study had a relatively larger age range, such that sleep patterns may differ and thus the effects of the intervention may have differed by age. Larger-scale randomized control trials that are powered for moderation analyses are needed to address this gap.

## Conclusions

Although counterintuitive, TiB restriction can be used in older adults to improve slow-wave and sleep efficiency components of sleep that are particularly problematic in older adults and have been shown to be associated with memory and cognitive function. TiB restriction is a technique that older adults can use in their own home without the risk of drug dependence and side effects or the need for specialized treatment or equipment to improve their sleep depth and sleep efficiency. A sufficient dose of behavioral TiB restriction arguably offers better ecological validity, accessibility, and feasibility optimal to existing SWA enhancement techniques. Longer-term and larger-scale studies will determine if improvements in memory arise after sleepiness dissipates and participants adjust to a sleep schedule that includes TiB restriction.

## Data availability statement

The raw data supporting the conclusions of this article will be made available by the authors, without undue reservation.

## Ethics statement

The studies involving humans were approved by University of Pittsburgh Institutional Review Board. The studies were conducted in accordance with the local legislation and institutional requirements. The participants provided their written informed consent to participate in this study.

## Author contributions

KW: Conceptualization, Formal analysis, Funding acquisition, Investigation, Methodology, Resources, Supervision, Validation, Writing—original draft. RH: Formal analysis, Investigation, Visualization, Writing—original draft. YD: Formal analysis, Visualization, Writing—original draft. MS: Formal analysis, Writing—review & editing. KD: Formal analysis, Writing—original draft. AW: Formal analysis, Writing—original draft. CP: Investigation, Methodology, Supervision, Writing— review & editing, Project administration. MF: Data curation, Methodology, Software, Writing—review & editing. DB: Conceptualization, Methodology, Resources, Supervision, Writing—review & editing.
