# Supplementary material for: A pilot time-in-bed restriction intervention behaviorally enhances slow-wave activity in older adults
Source: Front Sleep. Author manuscript; Available in PMC 2024 Jun 27. (PMC11210605; doi:10.3389/frsle.2023.1265006)
Supplement: Supplementary Material [file NIHMS2003344-supplement-Supplementary_Material.docx]

Supplementary Materials

Supplementary Figure 1: Karolinska Sleepiness Scale ratings in the TiB restriction group over the course of the week-long intervention. Higher values indicate higher levels of subjective sleepiness.


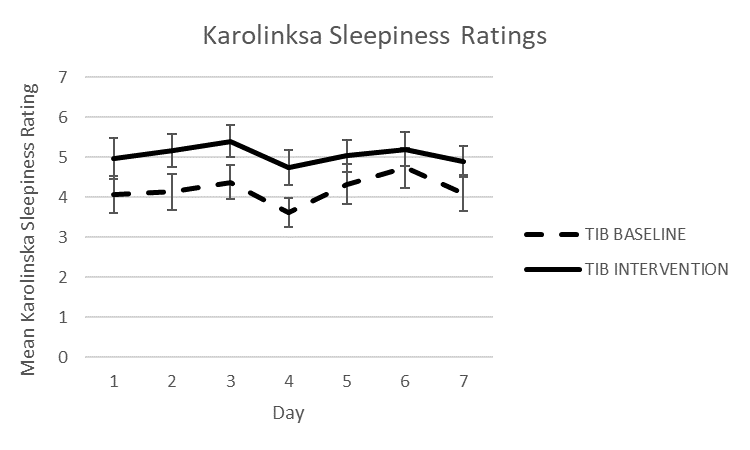


Supplementary Table 1: Means and standard deviations (in parentheses) for all primary and secondary measures.

|  | **TiB restriction Group** | | **Comparison Group** | |
| --- | --- | --- | --- | --- |
|  | Baseline | Intervention | Baseline | Intervention |
| **Epworth** | 6.3 (3.6) | 6.8 (5.0) | 8.4 (4.5) | 8.6 (5.8) |
| **ISI** | 12.7 (4.8) | 11.0 (5.1) | 11.0 (3.8) | 9.4 (3.8) |
| **TiB Diary** | 489.0 (62.9) | 386.1 (62.0) | 495.2 (83.6) | 542.4 (86.7) |
| **TiB Actigraphy** | 482.2 (62.7) | 365.0 (91.4) | 493.2 (103.7) | 520.2 (80.2) |
| **TiB PSG** | 489.6 (59.6) | 375.5 (52.1) | 528.1 (92.6) | 537.5 (82.3) |
| **TST Diary** | 337.1 (55.3) | 300.3 (40.5) | 355.0 (78.3) | 408.2 (57.2) |
| **TST Actigraphy** | 410.0 (51.7) | 328.9 (37.0) | 388.5 (112.3) | 437.6 (96.8) |
| **TST PSG** | 360.5 (79.4) | 318.0 (49.4) | 363.8 (25.5) | 411.9 (91.5) |
| **SE Diary** | 76.6 (11.6) | 84.2 (9.9) | 78.1 (6.0) | 83.5 (5.0) |
| **SE Actigraphy** | 83.7 (12.2) | 85.6 (11.7) | 75.7 (8.1) | 81.0 (7.4) |
| **SE PSG** | 73.7 (14.5) | 84.7 (8.8) | 70.6 (13.2) | 76.3 (11.0) |
| **WASO Diary** | 72.8 (41.2) | 30.2 (22.7) | 70.4 (20.8) | 49.8 (20.0) |
| **WASO Actigraphy** | 54.1 (23.2) | 37.5 (20.0) | 66.9 (24.4) | 60.6 (24.5) |
| **WASO PSG** | 102.3 (50.5) | 47.8 (27.6) | 140.8 (91.8) | 109.3 (52.8) |
| **Nap Mins** | 57.0 (77.7) | 24.7 (56.8) | 395.2 (273.3) | 68.8 (254.0) |
| **N1 Min** | 38.2(20.7) | 27.9 (15.1) | 35.0 (4.6) | 40.2 (17.0) |
| **N2 Min** | 209.7(53.3) | 183.1 (38.3) | 241.4 (31.6) | 269.0 (58.7) |
| **N3 Min** | 36.4 (38.7) | 35.2 (32.3) | 9(9.2) | 10.7 (10.8) |
| **REM Min** | 76.1 (28.5) | 71.8 (27.1) | 21.6(4.3) | 21.6(8.2) |
| **%N1** | 11.3 (7.2) | 8.8 (4.4) | 9.7 (1.6) | 10.1 (5.3) |
| **%N2** | 57.9 (7.5) | 57.8 (9.4) | 66.2 (4.7) | 65.7 (7.0) |
| **%N3** | 9.8 (9.7) | 11.4 (10.5) | 2.5 (2.8) | 2.5 (2.3) |
| **% REM** | 21.0 (6.0) | 22.0 (6.7) | 9.0(9.2) | 10.7(10.8) |
| **Absolute 0.5-1Hz** | 74.2(68.9) | 83.1(84.5) | 54.6(19.1) | 61.9(15.6) |
| **Absolute 1-4Hz** | 139.5(98.1) | 158.1(125.4) | 87.1(33.7) | 92.8(35.6) |
| **Relative 0.5-1Hz** | 25.79% (0.087) | 25.97% (0.074) | 30.70% (0.038) | 33.00% (0.06) |
| **Relative 1-4Hz** | 52.18% (0.073) | 52.95% (0.069) | 49.30% (0.024) | 48.60% (0.049) |
